# P-213. Nurse Experiences with a Universal Gloving Intervention to Prevent *Clostridioides difficile* Infection: A Qualitative Study

**DOI:** 10.1093/ofid/ofae631.417

**Published:** 2025-01-29

**Authors:** Linda McKinley, Julie Keating, Katherine Dolan, Helene Moriarty, Cara E Ray, Nasia Safdar

**Affiliations:** Wm. S. Middleton Memorial VA Hospital, Madison, Wisconsin; Madison VA Hospital, Madison, Wisconsin; University of Wisconsin - Madison, Madison, Wisconsin; Villanova University, Philadelphia, Pennsylvania; Edward Hines, Jr. VA Medical Center, Chicago, Illinois; University of Wisconsin School of Medicine and Public Health, Madison, WI

## Abstract

**Background:**

Gloving is recommended for healthcare workers caring for patients with *C. difficile* infections, however, the effectiveness of gloving for all patient contact (universal gloving) to prevent transmission from patients with asymptomatic C. difficile colonization is unknown. We conducted a clinical trial to evaluate a universal gloving intervention to prevent *C. difficile* acquisition among patients in VA acute care settings. We interviewed nurses at participating sites to understand their experiences with the intervention.

**Methods:**

We conducted 8 interviews and 4 focus groups across five participating intervention units at VA hospitals; 23 nurses participated in interviews and focus groups. We used a semi-structured interview guide to ask about the healthcare worker experience with universal gloving; facilitators and barriers to universal gloving; universal gloving impacts on hand hygiene, patient care, and workload; perceived patient experience with healthcare worker gloving; and perceived fidelity with the intervention. Interviews were audio recorded and transcribed, and we used rapid qualitative inquiry methods to analyze transcripts.

**Results:**

Participants reported that gloving was already a standard practice, and universal gloving was easier to expand as an existing practice rather than launching a new practice. Reported fidelity was mixed; some participants reported that gloving was unnecessary for some tasks and difficult to do 100% of the time such as when not having patient contact. Facilitators to universal gloving included providing demonstrated benefit to outweigh burdens, demonstrating leadership support, engaging full healthcare teams including other departments, and ensuring appropriate signage and glove availability. Barriers to universal gloving included role-dependent variation in awareness and compliance, communication across roles, and impacts to patient care such as with tactile tasks and emergency responses.

**Conclusion:**

While universal gloving did not require major changes to nurse workflow or training because of its use for standard and transmission-based precautions, there were increased role- and task-dependent barriers to implementation of universal gloving for all patient contact.

**Disclosures:**

**All Authors**: No reported disclosures

